# Estrogen-induced immune changes within the normal mammary gland

**DOI:** 10.1038/s41598-022-21871-4

**Published:** 2022-11-08

**Authors:** Helen Tower, Genevieve Dall, Ashleigh Davey, Melanie Stewart, Patrick Lanteri, Meagan Ruppert, Maria Lambouras, Ibraheem Nasir, Serene Yeow, Phillip K. Darcy, Wendy V. Ingman, Belinda Parker, Nicole M. Haynes, Kara L. Britt

**Affiliations:** 1 Breast Cancer Risk and Prevention Laboratory, Peter MacCallum Cancer Centre, 305 Grattan St, Melbourne, VIC 3000 Australia; 2 The Walter and Eliza Hall Institute of Medical Research, Parkville, VIC Australia; 3 Structural Biology Division, The Walter and Eliza Hall Institute of Medical Research, Parkville, 5052 Australia; 4 Department of Anatomy and Developmental Biology, Monash University Clayton, Wellington Rd, Clayton, 3800 Australia; 5 Sir Peter MacCallum Department of Oncology, The University of Melbourne, Parkville, Melbourne, VIC Australia; 6 Cancer Immunology Program, Peter MacCallum Cancer Centre, Melbourne, Australia; 7 Discipline of Surgical Specialties, Adelaide Medical School, The Queen Elizabeth Hospital, University of Adelaide, Adelaide, SA 5011 Australia; 8 Robinson Research Institute, University of Adelaide, Adelaide, SA 5005 Australia; 9 Cancer Evolution and Metastasis Program, Peter MacCallum Cancer Centre, Melbourne, VIC Australia; 10 Cancer Therapeutics Program, Peter MacCallum Cancer Centre, Melbourne, VIC Australia

**Keywords:** Cancer, Cell biology, Immunology

## Abstract

Breast cancer (BCa) incidence increases following aberrant hormone exposure, which has been linked to direct effects on estrogen receptor (ER)^+^ mammary epithelium. While estrogen exposure during mammary involution has been shown to drive tumour growth via neutrophils, the potential for the ER + immune microenvironment to mediate part (in addition to mammary epithelial cells) of hormonally controlled BCa risk during normal development has not been assessed. We collected mammary tissue, lymph nodes and blood from tumour naïve mice treated with, oophorectomy, estrogen (17β estradiol) or Fulvestrant. Flow cytometry was used to examine the impact on the frequency of innate and adaptive immune cells. Oophorectomy and fulvestrant decreased the proportion of macrophages, particularly pro-tumour polarized M2 macrophages and neutrophils. Conversely, dendritic cells were increased by these therapies, as were eosinophils. Estrogen increased the proportion of M2 macrophages and to a lesser extent CD4-CD8- double negative and FoxP3^+^ regulatory T cells but decreased CD8 + T cells and B cells. Excluding eosinophils, these changes were restricted to the mammary tissue. This suggests that inhibiting estrogen action lowers the immune suppressive myeloid cells, increases in antigen presentation and eosinophil-mediated direct or indirect cytotoxic effects. In contrast, estrogen exposure, which drives BCa risk, increases the suppressive myeloid cells and reduces anti-tumour cytotoxic T cells. The impact of hormonal exposure on BCa risk, may in part be linked to its immune modulatory activity.

## Introduction

Therapies that block or deplete estrogen are currently the only approved breast cancer (BCa) preventatives. Hormonal manipulation has also been the cornerstone of BCa treatment following the discovery in the nineteenth century that an ovariectomy could decrease BCa size. This gave rise to endocrine therapies such as tamoxifen and raloxifene (selective estrogen receptor (ER) modulators) that block estrogen binding of the ER and aromatase inhibitors that block estrogen production. Both therapeutic approaches have been demonstrated to reduce BCa incidence^1^. However, tamoxifen is the only preventive agent that has proven effective in both premenopausal and postmenopausal women, reducing the risk of ER+ positive BCa risk by 33%^2^. Furthermore, pre-clinical prevention experiments in mice have shown that BCa growth is inhibited by both oophorectomy and fulvestrant^3–5^, whilst estrogen can stimulate tumorigenesis ^6^.

ER expression in both the nucleus and cytoplasm of normal breast epithelial cells and tumour cells allows estrogen-induced signalling pathways to stimulate morphogenesis and ductal outgrowth in the normal breast and tumour cell survival and proliferation in cancer. We have shown that ER alpha expression within the mammary epithelial cells correlates with estrogen responsiveness and proliferation^7^ and others have shown breast development is blocked in the absence of ER alpha^8^. Thus, the preventative impact of blocking estrogen action has long been thought to be a direct effect via ER^+^ breast epithelial cells. However, it is now clear that ERs are expressed on numerous immune cells including macrophages, DCs, B cells and T cells^9^. In healthy breast tissue, immune populations are modulated during the ovarian cycle as well as during pregnancy and lactation^10^. Whilst the effects of estrogen on cytokine secretion are specific to the cell type, conditions within the organ and concentration of estrogen, ovarian hormones and estrogens have been shown to increase the cytokine abundance in the mammary gland^11,12^. Within cancer, estrogen can drive an immunosuppressive tumour microenvironment that in fact stimulates tumour growth^12^. Specifically, mouse studies have shown that estrogen promotes an influx of M2-polarised macrophages into breast tumours^13^. M2 macrophages (anti-inflammatory or alternatively activated) have pro-tumour functions – they can induce fibrosis, the production of matrix, trigger angiogenesis and suppress T cell activity. Estrogen also increases the percentage and number of immunosuppressive myeloid-derived suppressor cells in ovarian cancer whilst decreasing the number of tumour-associated helper and cytotoxic T cells^14^. Estrogen also appears to weigh the tumour immune balance towards tumour-promoting cytokines (IL-6, IL-4, TNF and IL-17A), M2 (pro-tumour) macrophage polarisation and diminished functional capacities of anti-tumour NK and CD8^+^ T cells^12^. This is accompanied by proliferation of immuno-inhibitory T regulatory cells and increased expression of immune checkpoint ligand PD-L1.

Emerging data suggest that endocrine therapies may exert their effects, at least in part, through modulation of the immune microenvironment of the breast. Endocrine therapies are approved to lower the risk of contralateral BCa in BRCA1 and BRCA2 mutation carriers^15^. When the efficacy was assessed, tamoxifen was found to protect against both ER + and ER- tumour development^15^. Similarly, ovariectomy can delay tumorigenesis in mice implanted with aggressive ER-negative ovarian cancer cells^14^, but these effects are lost in immune-compromised mice. Oophorectomy and anti-estrogens have been shown to reduce the progression of 4T1 triple negative breast cancers^16^ and improve the efficacy of checkpoint inhibitor therapy. Interestingly, in a high throughput drug screen, the anti-estrogen fulvestrant was identified as one of the top drugs to sensitise lung cancer cells to immune mediated cell death^17^. What remains unclear, is whether estrogens and anti-estrogens can influence the local mammary immune landscape prior to tumour development and thus in part mediate the risk conferred by these exposures^1^. Support that this may occur has recently been provided by work showing that during mammary gland involution (which follows pregnancy) estrogen supplementation can drive tumour growth through increased neutrophil recruitment^18^.

Here, we show exogenous estrogen elicited immunosuppressive changes to the innate and adaptive immune microenvironment that may explain the increased risk of BCa following estrogen exposure. In contrast, eliminating estrogen activity via oophorectomy or fulvestrant treatment promoted an increase in immune cells considered more tumour cytotoxic and tumour suppressive.

## Results

To assess the impact of hormone exposure on the immune microenvironment of mouse mammary tissue, mice were either oophorectomized, treated for 6 weeks with 17β estradiol or the anti-estrogen Fulvestrant. Wildtype untreated mice were used as controls. To confirm that the hormonal manipulations were effective, we assessed uterine wet weight as the uteri are highly sensitive to estrogen supplementation^19^. Oophorectomy reduced the uterine weights as did anti-estrogen treatment (p < 0.0001), whilst estradiol led to an increase in uterine weight of the mice (p < 0.05) Supplementary Table 3.

Similar trends were also observed for mammary gland weight in response to each of the treatments, although to a lesser extent.

### Estrogen impacts the innate immune microenvironment of the non-neoplastic mammary gland

**Figure 1:**
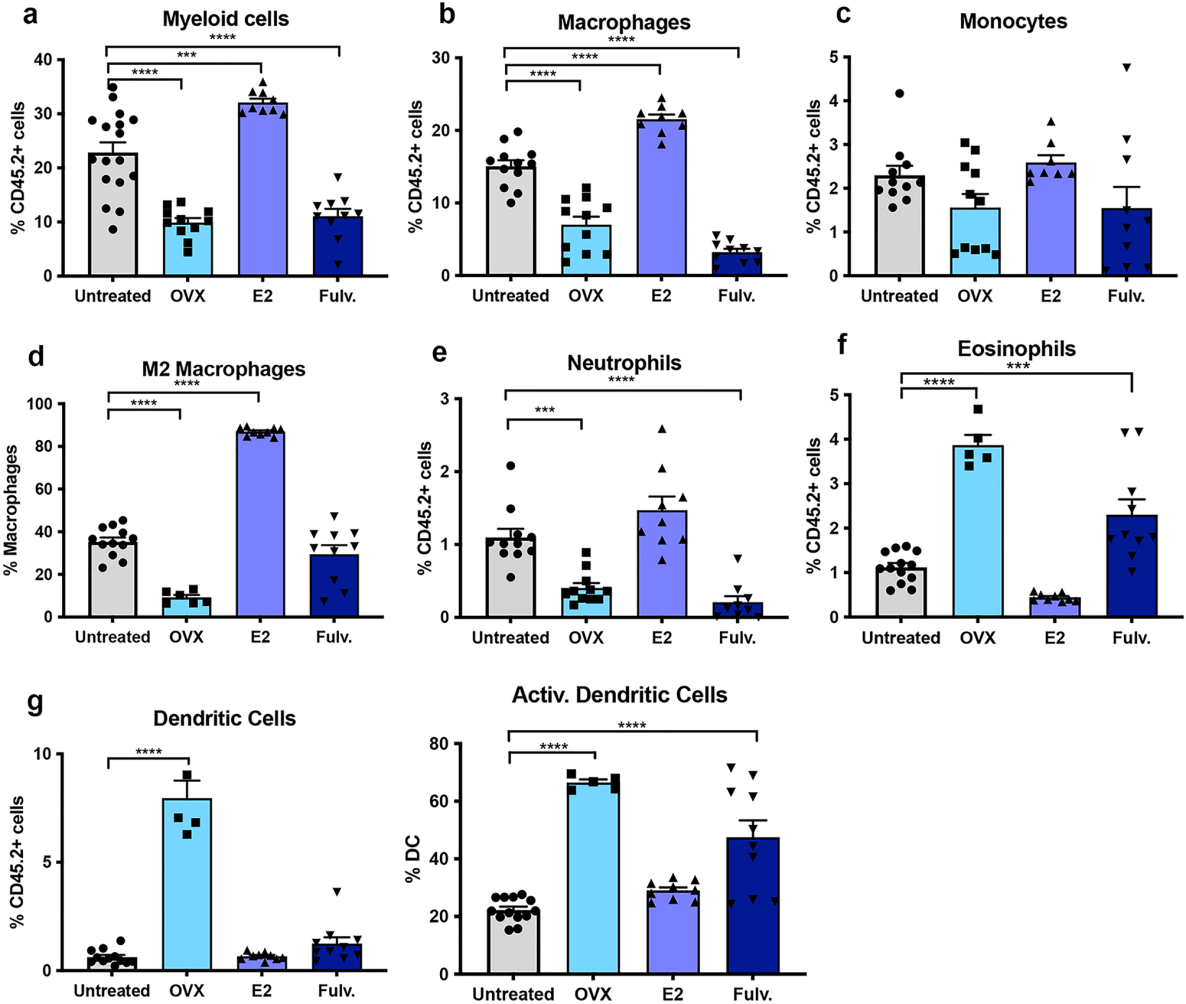
Effects of estrogen, oophorectomy and fulvestrant on the innate immune cells within the non-neoplastic mouse mammary gland. Mice were either untreated, ovariectomised (Ooph) or treated for 6 weeks with estrogen (E2) or Fulvestrant (Fulv). (**a**) Myeloid cells, (**b**) macrophages, (**c**) monocytes, (**d**) M2 macrophages, (**e**) neutrophils, (**f**) eosinophils, (**g**) dendritic cells. Results are expressed percentage of immune cells (mean ± SEM). n = 11–17 mice/group. Data was analysed using a Turkey multiple comparisons one-way ANOVA if confirmed Gaussian distribution, or Kruskal–Wallis multiple comparisons 1-way ANOVA if non-Gaussian. Statistically significant results are denoted with *p < 0.05, **p < 0.01, ***p < 0.001, ****p < 0.0001.

As the impact of hormonal exposure on the immune cells within the normal mammary gland have not been assessed we measured the innate and adaptive immune cells on a per mouse basis. The percentage of mammary associated myeloid cells was significantly increased with estrogen treatment when compared to untreated controls, whilst oophorectomy and fulvestrant induced a decrease in myeloid cells compared to untreated mice (Fig. 1a). Within the myeloid compartment, estrogen increased the proportion of macrophages, many of which expressed CD206, indicative of a pro-tumorigenic M2 phenotype but did not impact the monocytes (Fig. 1b–d). A similar trend was observed for neutrophils (p = 0.08) (Fig. 1e). In contrast, oophorectomy and fulvestrant caused a significant decrease in macrophage and neutrophil numbers (Fig. 1b,e), but only oophorectomy led to a decrease in CD206^+^ M2 macrophages (Fig. 1d). Of the treatments analysed oophorectomy and fulvestrant significantly increased the proportion of mammary associated eosinophils and oophorectomy the dendritic cells (Fig. 1f,g).

### Estrogen impacts on the adaptive immune microenvironment of the non-neoplastic mammary gland

**Figure 2:**
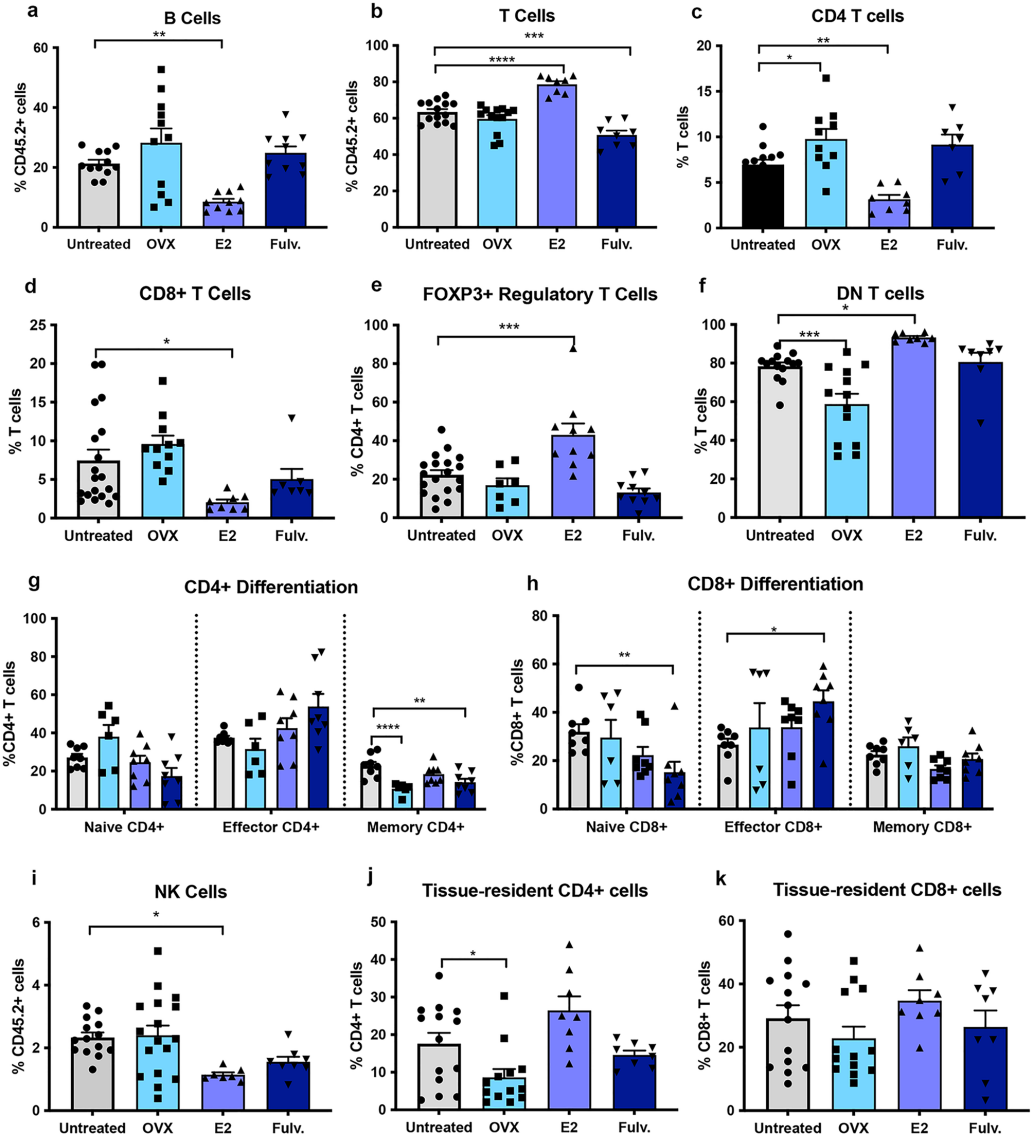
Effects of estrogen, oophorectomy and fulvestrant on the adaptive immune cells within the non-neoplastic mouse mammary gland. Mice were either untreated, ovariectomised (Ooph) or treated for 6 weeks with estrogen (E2) or Fulvestrant (Fulv). (**a**) B cells, (**b**) T cells, (**c**) CD4 + T helper cells, (**d**) CD8 + cytotoxic T cells, (**e**) FoxP3 + regulatory T cells, (**f**) CD4- CD8- T cells, (**g**) CD4 + differentiation status, (**h**) CD8 + differentiation status, (**i**) NK cells, (**j**) tissue resident CD4 + T cells and (k) tissue resident CD8 + T cells. Results are expressed percentage of immune cells (mean ± SEM). n = 11–17 mice/group. Data was analysed using a Turkey multiple comparisons one-way ANOVA if confirmed Gaussian distribution, or Kruskal–Wallis multiple comparisons 1-way ANOVA if non-Gaussian. Statistically significant results are denoted with *p < 0.05, **p < 0.01, ***p < 0.001, ****p < 0.0001.

Exogenous estrogen treatment reduced the frequency of mammary B cells (Fig. 2a) whilst increasing the proportion of T cells (Fig. 2b). FOXP3^+^ T regulatory and CD4^-^CD8^-^ double-negative T cells were the dominant T cell subsets detected post estrogen treatment. The proportion of CD4^+^ and CD8^+^ T cells was reduced relative to the untreated controls (Fig. 2c–f). Notably the phenotype of the CD4 and CD8 T cell compartments remained relatively unchanged. In the case of oophorectomy, a reduction in CD4^+^ central memory cells was observed and fulvestrant treatment resulted in a reduction of naïve CD8 T cells but increased CD8^+^ T effector cells (Fig. 2g,h). NK cells whilst bridging innate and adaptive immune cells were assessed as part of our adaptative panel. The proportion of NK cells as a percentage of all immune cells were decreased by estrogen treatment (Fig. 2i) but not significantly altered by oophorectomy or fulvestrant. We also assessed the activated NK cells using CD69, but did not find any impact of hormonal exposure on the degree of activation (Supplementary Fig. 2). The frequency of issue resident CD4^+^ and CD8^+^ T cells was not significantly altered by oophorectomy, estrogen or fulvestrant, compared to untreated controls (Fig. 2j–k).

### Estrogen effects the immune composition of intra mammary lymph nodes

**Figure 3:**
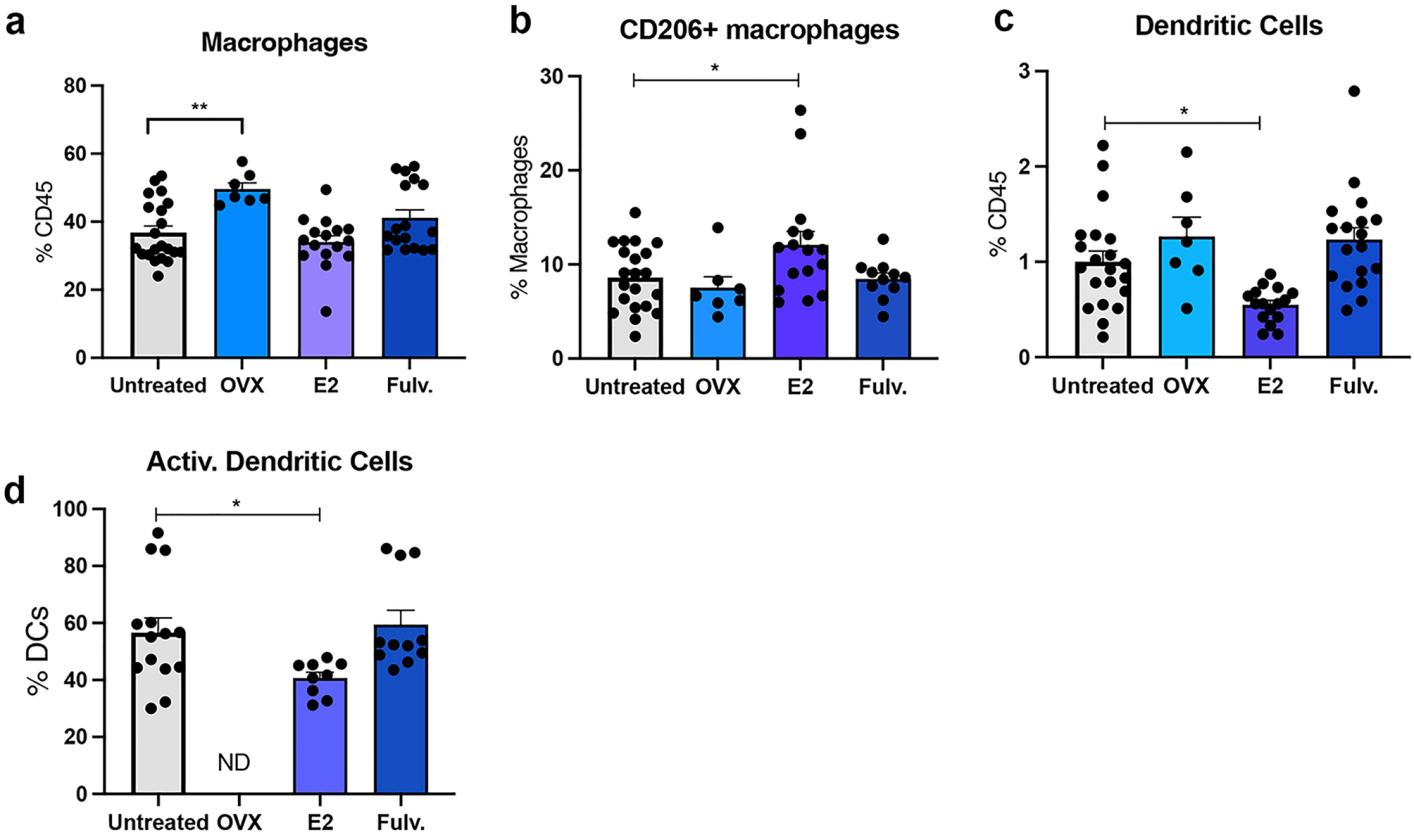
Effects of estrogen, oophorectomy and fulvestrant on the mammary lymph node immune cells. Mice were either untreated, ovariectomised (Ooph) or treated for 6 weeks with estrogen (E2) or Fulvestrant (Fulv). (**a**) macrophages, (**b**) CD206 + macrophages, (**c**) dendritic cells (**d**) activated (CD86 +) dendritic cells. Results are expressed percentage of immune cells (mean ± SEM). n = 7–21 mice/group. Data was analysed using a Turkey multiple comparisons one-way ANOVA if confirmed Gaussian distribution, or Kruskal–Wallis multiple comparisons 1-way ANOVA if non-Gaussian. Statistically significant results are denoted with *p < 0.05, **p < 0.01.

Lymph nodes are important in the anti-tumour response as naive T cells interact with antigen presenting cells (APCs) to generate T cell-dependent immune responses. The lymph nodes showed some changes, mainly with estrogen, but not with oophorectomy or fulvestrant. Analysis of the myeloid compartment of the inguinal lymph node revealed that estrogen did not affect the overall frequency of lymph node associated macrophages (Fig. 3a), but it did selectively increase the proportion of CD206^+^ macrophages (Fig. 3b). Oophorectomy increased in the proportion of macrophages but the CD206 + M2 macrophages remained unchanged (Fig. 3a–b). Fulvestrant did not change the macrophage populations. Estrogen led to a reduction in the total DC frequency, as well as activated DCs (Fig. 3c,d), but oophorectomy and fulvestrant did not impact them.

**Figure 4:**
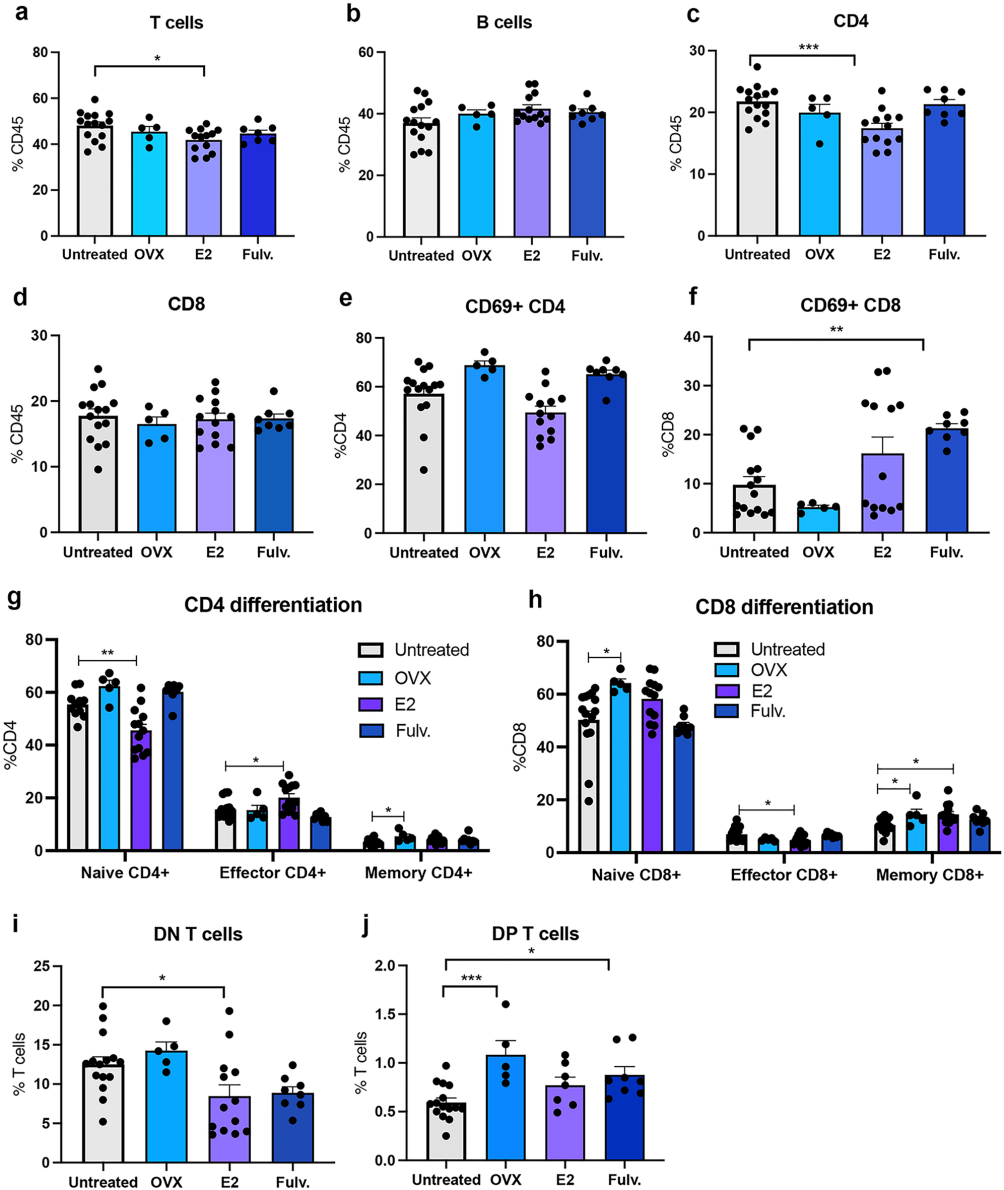
Effects of estrogen, oophorectomy and fulvestrant on the mammary lymph node immune cells. Mice were either untreated, ovariectomised (Ooph) or treated for 6 weeks with estrogen (E2) or Fulvestrant (Fulv). (**a**) T cells, (**b**) B cells, (**c**) CD4 + T helper cells, (**d**) CD8 + cytotoxic T cells, (**e**) Activated CD4 + T cells, (**f**) activated CD8 + T cells, (**g**) CD4 + differentiation status, (**h**) CD8 + differentiation status, (**i**) CD4- CD8- T cells, (**j**) CD4 + CD8 + T cells. Results are expressed percentage of immune cells (mean ± SEM). n = 7–21 mice/group. Data was analysed using a Turkey multiple comparisons one-way ANOVA if confirmed Gaussian distribution, or Kruskal–Wallis multiple comparisons 1-way ANOVA if non-Gaussian. Statistically significant results are denoted with *p < 0.05, **p < 0.01, ***p < 0.001.

Within the adaptive immune system, estrogen led to lower levels of T cells in the lymph node, but did not alter the proportion of B cells. Oophorectomy and fulvestrant did not alter them (Fig. 4a–b). CD4 T cells were decreased by estrogen but total CD8s were not impacted (Fig. 4c–d). Oophorectomy and fulvestrant did not impact the lymph node CD4 and CD8 T cells. Estrogen did not impact the proportion of activated CD4 or CD8 T cells (Fig. 4e–f) but fulvestrant increased the number of activated CD69 + CD8 T cells (Fig. 4f). Estrogen increased the proportion of CD4 central memory effector cells and decreased CD8 central memory effectors (Fig. 4g–h). Oophorectomy increased the CD4 central memory CD4 T cells. Oophorectomy increased the proportion central memory CD8^+^ T cells, and naïve CD8^+^ T cells (Fig. 4h). Estrogen led to increased CD8 central memory and effector CD8 T cells (Fig. 4h). CD4^-^CD8^-^ double-negative T cells were significantly lost with estrogen exposure (Fig. 4i) whilst oophorectomy and fulvestrant increased CD4 + CD8 + double-positive T cells (Fig. 4j).

### Different effects of estrogen on systemic immune cell composition compared to the mammary tissue

**Figure 5:**
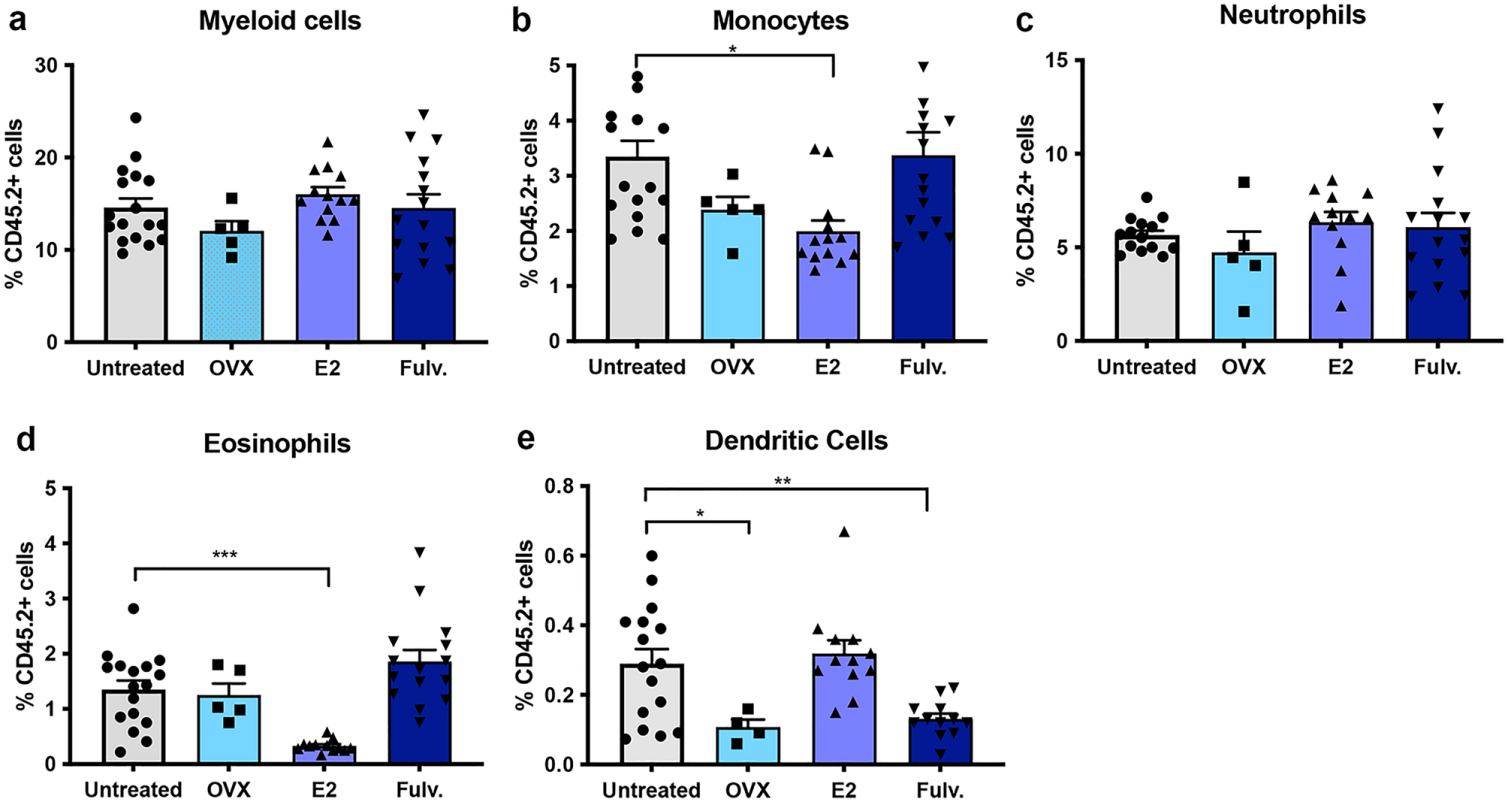
Effects of estrogen, oophorectomy and fulvestrant on the blood innate immune cells. Mice were either untreated, ovariectomised (OVX) or treated for 6 weeks with estrogen (E2) or Fulvestrant (ICI). (**a**) Myeloid cells, (**b**) monocytes, (**c**) neutrophils, (**d**) eosinophils, (**e**) dendritic cells. Results are expressed percentage of immune cells (mean ± SEM). n = 5–17 mice/group. Data was analysed using a Turkey multiple comparisons one-way ANOVA if confirmed Gaussian distribution, or Kruskal–Wallis multiple comparisons 1-way ANOVA if non-Gaussian. Statistically significant results are denoted with *p < 0.05, **p < 0.01, ***p < 0.001, ****p < 0.0001.

Interestingly, within the blood the innate immune cells were not impacted as much by hormonal exposure as they were in the mammary gland. The striking induction of DCs in the mammary tissue with oophorectomy was not evident in the blood. Instead, oophorectomy and fulvestrant decreased systemic DCs. Monocytes were slightly decreased by estrogen. Neutrophils, which were decreased by oophorectomy and fulvestrant in the mammary gland, were not altered systemically (Fig. 5).

**Figure 6:**
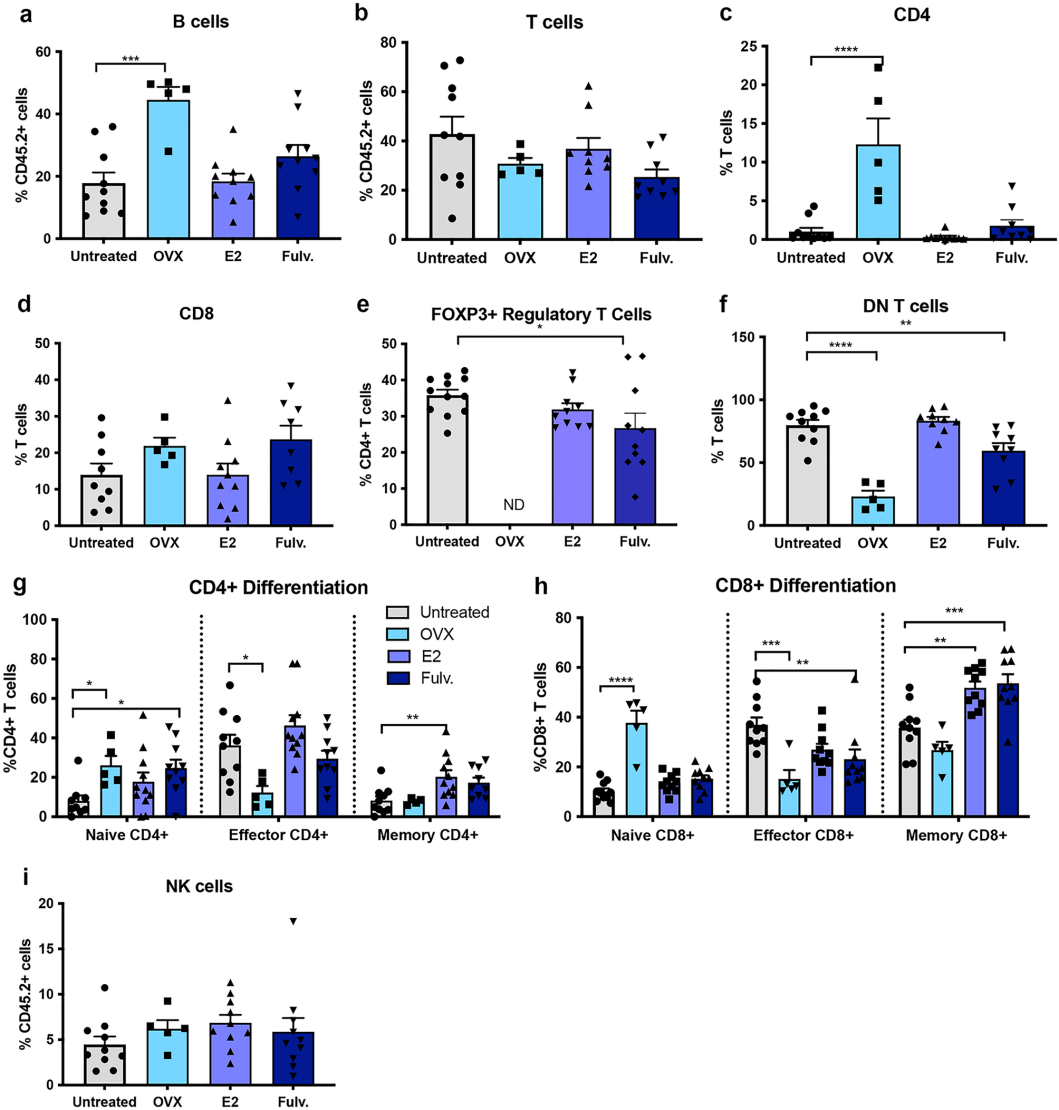
Effects of estrogen, oophorectomy and fulvestrant on the blood adaptive immune cells. Mice were either untreated, ovariectomised (Ooph) or treated for 6 weeks with estrogen (E2) or Fulvestrant (Fulv). (**a**) B cells, (**b**) T cells, (**c**) CD4 + T helper cells, (**d**) CD8 + cytotoxic T cells, (**e**) FoxP3 + regulatory T cells, (**f**) CD4- CD8- T cells, (**g**) CD4 + differentiation status, (**h**) CD8 + differentiation status and (**i**) NK cells. Results are expressed percentage of immune cells (mean ± SEM). n = 5–17 mice/group. Data was analysed using a Turkey multiple comparisons one-way ANOVA if confirmed Gaussian distribution, or Kruskal–Wallis multiple comparisons 1-way ANOVA if non-Gaussian. Statistically significant results are denoted with *p < 0.05, **p < 0.01, ***p < 0.001, ****p < 0.0001.

Within the adaptive immune system some of the trends observed in the mammary gland, were seen systemically, but not all. In contrast to the mammary gland, B cells were only altered by oophorectomy and T cells were unchanged (Fig. 6a,b). CD4 T cells were increased and CD4^−^ CD8^−^ double negative T cells were decreased by oophorectomy but FoxP3 cells were not impacted, and CD8 T cells were unchanged by any treatment (Fig. 6c–f). FoxP3 T cells were instead decreased by fulvestrant (Fig. 6e). However, whilst the mammary gland showed minimal changes to the central memory CD4 and CD8 T cells, changes were seen systemically. Naive CD4 T cells were increased by oophorectomy and fulvestrant and effector CD4 T cells were reduced by oophorectomy. Estrogen treatment led to increased circulating memory CD4 T cells (Fig. 6g). CD8 central memory cells showed increased naïve cells and decreased effectors in oophorecotmised and in fulvestrant treated mice (Fig. 6h). Memory cells were increased by estrogen and fulvestrant treatment. NK cells were decreased by estrogen in the mammary gland, but not impacted systemically (Fig. 6i).

### Mammary epithelial cell cytokine expression in response to estrogen exposure

**Figure 7:**
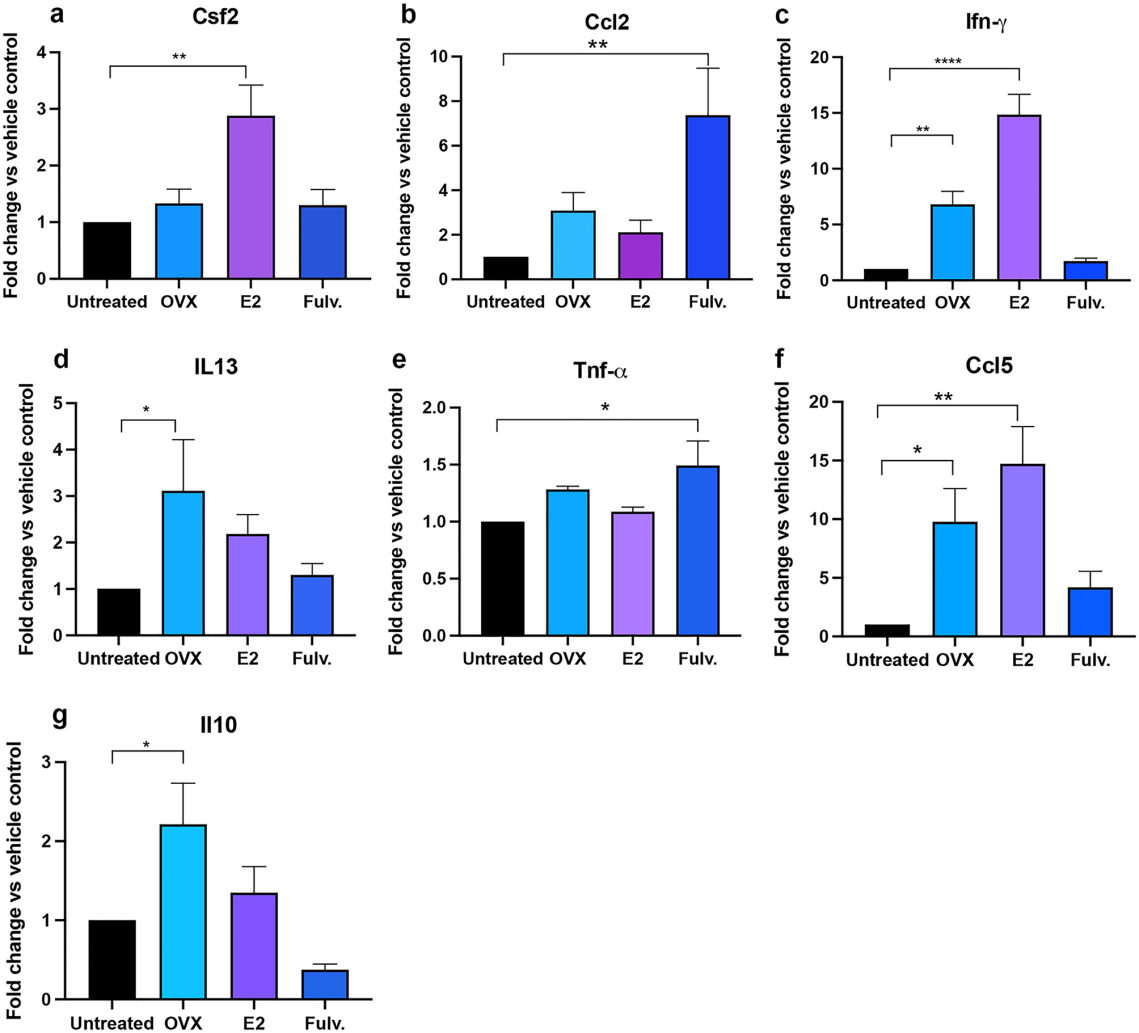
Effects of estrogen, oophorectomy and fulvestrant on transcript expression of myeloid specific cytokines in the mouse mammary epithelial cells. Mice were either untreated, ovariectomised (Ooph) or treated for 6 weeks with estrogen (E2) or Fulvestrant (Fulv). (**a**) Csf-2, (**b**) Ccl2, (**c**) Ifn-α, (**d**) IL-13, (**e**) Tnf-α, (**f**) Ccl5, (**g**) Il-10. Results are expressed percentage of immune cells (mean ± SEM). n = 4 biological replicates of n = 2–10 mice/group. Data was analysed using a Turkey multiple comparisons one-way ANOVA if confirmed Gaussian distribution, or Kruskal–Wallis multiple comparisons 1-way ANOVA if non-Gaussian. Statistically significant results are denoted with *p < 0.05, **p < 0.01, ***p < 0.001, ****p < 0.0001.

While immune cells express ER, many of the effects we observed were specific to the mammary gland and not observed systemically, indicating that the mammary epithelial cells, which are known to express higher levels of ER^20^ may be contributing to the changes. To ascertain this, we treated a second set of mice with estrogen, oophorectomy or fulvestrant and isolated the EpCAM^+^ mammary epithelial cells by FACs 6 weeks after treatment. Real time quantitative PCR (qPCR) was performed on the epithelial cells to quantitate the level of expression of numerous cytokines. With limited material we focussed on myeloid specific cytokines. In line with the increase in mammary macrophages and neutrophils with estrogen treatment Csf2 and Ccl5 expression in the mammary gland were increased by estrogen. Oophorectomy increased IL-13 and Ccl5 which recruit eosinophils, and this aligned with the increased eosinophils in oophorectomized mammary glands. Fulvestrant increased Ccl2 and Tnf which we expect may have increased the myeloid cells but this was not observed potentially indicating these more modest increases were not biologically relevant (Fig. 7). Il-4, Csf1r and Tgfb were assessed but showed similar expression across groups.

## Discussion

Oophorectomy and endocrine therapies are currently used to prevent BCa. Whilst work is ongoing to determine if the epithelial cells within the non-tumorigenic epithelium are altered by these therapies (Dall et al. unpublished), it is clear that hormonal exposure impacts the normal mammary epithelium^21–23^ which is known to expresses high levels of estrogen receptor. However, the efficacy endocrine therapies in prevention and treatment is not restricted to hormonally driven ER^+^ BCa. In the treatment space, endocrine therapy still provides some benefit to ER negative breast and ovarian tumours, effects which are lost in immune deficient mouse models^14,16^ indicating the immune system may play a role. Thus, in addition to the ER + epithelium, some of the anti-tumour benefit from preventative endocrine therapies may be mediated via the ER^+^ immune microenvironment. We show that oophorectomy and fulvestrant lead to a decrease in macrophages and neutrophils in the mouse mammary gland (which have been associated with T cell suppressive activity), and an increase in antigen presenting dendritic cells and eosinophils. In contrast, estrogen was associated with elevated levels of suppressive myeloid cells and fewer anti-tumour cytotoxic T cells. This is the first evidence that the proportion of immune cells in the non-neoplastic mammary gland is altered by surgery, chemo-preventatives and exogenous estrogen exposure. Our data also indicates that exogenous estrogen exposure (for instance, oral contraceptives, hormone replacement therapy and environmental estrogens) may increase BCa risk by direct effects on the local immune microenvironment, in addition to the well characterised pro-proliferative effects on the epithelium.

BCa has numerous subtypes including triple negative BCa (TNBC), which lack ER, PR and HER2. In TNBC tumours, the presence of tumour infiltrating lymphocytes (TILs) correlates with good prognosis and good response to chemotherapy. TILs have also been found to be a prognostic indicator for higher rates of pathological complete responses (pCRs) to neoadjuvant chemotherapy^24–27^. Twenty to thirty percent of TNBC patients respond to immunotherapy such as immune checkpoint inhibitors (ICI)^28,29^, and ICI have recently become available for advanced BCa patients. More recently, pre-clinical experiments have shown that endocrine therapies such as Fulvestrant have anti-tumour effects in ER- tumours in-vivo, which are dependent on the immune system^14^. Additionally, endocrine therapies have been shown to improve the efficacy of ICI in TNBC tumours in mice^16^. This raises the possibility that preventative endocrine therapy may also exert some of its action via immune mechanisms. Studies pre-treating mice and then challenging with tumours will allow the impact of these hormonal manipulations on the immune response to cancer initiation to be determined.

Our data show that currently approved preventive therapies, oophorectomy and endocrine therapy^1^, lead to a decrease in macrophages, and in particular a reduction in pro-tumour M2 macrophages. We have shown macrophages are increased in women at risk of developing BCa due to high mammographic density^30^. Similarly, macrophages are increased in benign prostatic hyperplasia^31^ and during hyperplasia of the endometrium^32^. Macrophages exist within many possible activation states in response to growth factors and external cues. Whilst an oversimplification, these states can be broadly divided on a spectrum ranging from M1 tumour-inhibiting macrophages to M2 tumour-promoting macrophages^33^. In the mouse mammary gland, M1 macrophages are associated with reduced cancer susceptibility, and reduction in this macrophage subtype due to endogenous TGFβ signaling increases cancer risk^34^. As M2 macrophages and neutrophils can both stimulate angiogenesis, cancer stem cell niche remodelling and cytotoxic T cell inhibition, it is plausible that these changes induced by oophorectomy and endocrine therapy provide the tissue with an improved ability to respond to early tumour growth and transformation.

We saw an increase in antigen presenting dendritic cells which can be thought of as tumour protective due to their ability to process and present antigens to T and B cells to generate an immune response^35^. Depletion of DCs using CD11c-DTR transgenic mice leads to increased tumour growth^36^, and DCs have been shown to increase in ER negative 4T1 tumors that are responding to endocrine therapies^16^. Similarly in the clinic, CDK4/6 inhibitors in combination with hormone therapy are being used to treat hormone receptor-positive (HR +), HER2-negative metastatic BC^37,38^. CDK4/6 inhibitors target proteins called cyclin dependent kinases 4 and 6 which regulate progression of the cell cycle. In addition to cell cycle arrest in the cancer cells, these drugs have been shown to drive enhanced antigen presenting capabilities of macrophages and DCs via upregulating MHC class I and II^39^.

Eosinophils are most known for their role in allergic inflammation; however, they have large granules, which store a variety of preformed cytokines/chemokines that are all cytotoxic. Once activated, eosinophils rapidly release their cytotoxic contents, which in turn may induce tissue remodeling and direct kill tumor cells^40–42^. Eosinophil numbers are associated with improved prognosis within numerous solid tumors^43^ and have recently been shown in melanoma to enhance the infiltration of cytotoxic T-cells^44^.

In contrast to the beneficial immune changes induced by endocrine therapy and oophorectomy, estrogen exposure (which is linked to enhanced BCa risk) increased immune cell suppressive myeloid cells and decreased the levels of anti-tumour cytotoxic T cells. BCa patients with higher M2 macrophages are associated with poorer survival^45,46^ which agrees with our findings. Estrogen treatment also decrease the levels of DN T cells. The function of these unconventional DN T cells remains largely unknown. DN T cells still retain their TCR composed of $\alpha \beta$ chains and have been shown to use TCR engagement as a means of initiating CD8 + T cell apoptosis^47^ and so a decrease in DN T cells would lead to a reduction in the repressive activity on CD8 T cells. This aligns with our data showing that estrogen increased CD8 T cells.

Many of the effects we saw following endocrine therapy were not observed systemically, leading us to postulate that the strong effects on the innate immune cells may have been in part mediated by signals from the mammary epithelial cells and the chemokines they produce. The normal mammary gland is poised to do this as it recruits and activates immune cells during its initial development, during puberty and within the pregnancy/lactation/involution cycle. If specific immune cell populations are blocked, normal breast development is impacted^48,49^. Supporting our work with endocrine therapy and oophorectomy, estrogen deprived BCa cells with increased levels of chemokines compared to non-deprived cells, enhance immune cell migration^50^. Using qPCR on epithelial cells isolated from treated mammary glands we found that transcript for Csf2, Interferon-γ (Ifn-γ) and Ccl5 were increased by estrogen exposure. Colony Stimulating Factor 2 (Csf2), also referred to as Granulocyte–macrophage colony-stimulating factor (G-csf), stimulates the survival, proliferation and differentiation of hematopoietic myeloid cells^51,52^. This aligns with the increased levels of myeloid cells, macrophages and to some extent neutrophils following estrogen treatment. Similarly, Ccl5 promotes the recruitment of T lymphocytes and macrophages^53,54^. In addition to direct effects on the mammary immune cells, additional signals from the estrogen sensitive epithelium is likely driving the larger impact on local compared to systemic immune changes.

Oophorectomy increased IFN-γ, Il-13, Ccl5 and Il-10, while fulvestrant increased Ccl2 and Tnf-α. Some of these differences align with the immune cell abundance in the mammary gland. For example Il-13 and Ccl5 stimulate eosinophils and oophorectomy had much higher levels of eosinophils than any other group. However, the increase in Il-10 and Tnf did not align with fulvestrant specific changes to T cells. We also did not assess a wide enough array of cytokines (due to limiting RNA) to define the DC changes in the oophorectomized treated mice.

Ifn-γ stimulates the anti-tumor immune response and possesses cytostatic, pro-apoptotic, anti-proliferative and anti-angiogenic properties^55^. Increased Ifn-γ following oophorectomy is consistent with its ability to mitigate BCa risk. However, increased levels in estrogen treated mice seems at odds with what we know about this tumoricidal cytokine. Estrogen can drive Ifn-γ gene expression in lymphocytes via estrogen response elements^56^ and as its aberrant production can drive autoimmunity Ifn-γ production is tightly controlled. It is possible the different hormonal milieu is mediating different downstream effects of this cytokine. IL-13 promotes survival, activation, and recruitment of eosinophils^57–59^ and thus increased levels in the mammary epithelium of oophorectomized mice is likely driving the increased mammary eosinophils. CCL-5 can stimulate recruitment of macrophages and T cells which may explain the changes to T cells observed with oophorectomy. IL-10 has can induce CD8^+^ T cell cytotoxicity^60^ and tumour rejection in preclinical tumour models^61^, and thus may assist in mediating the protective effects of oophorectomy. TNF-α can exert both tumour-inhibitory or tumour-promoting effects by inducing apoptosis or necroptosis, cell growth, cellular invasion or propagation of cancer cells. It can increase the expression of COX2, IL6 and IL8, whilst also reducing collagen synthesis and deposition in breast fibroblasts^62^. The pro and anti-tumour roles of TNF-α have largely been deduced from cancer studies, and further work on the cancer free breast may reveal the downstream impact of elevated TNF-α following fulvestrant treatment^63^. Fulvestrant treatment also increased CCL-2 (monocyte chemoattractant protein-1), which increases macrophage infiltration and susceptibility to cancer^64^. Ccl2 is also required for the immunosurveillance of small and/or developing tumours and may play a role in tumour progression in established cancers^65^. We acknowledge our epithelial analysis was restricted to PCR, and in the future cytokine bead arrays may help define whether the transcript changes translate to protein.

Whilst limited work exists for the assessment of lymph node response to endocrine therapy, recent findings indicate that the immune balance in the tumour-draining regional lymph node is significantly involved in anti-cancer immune responses. M1-polarised macrophages in the lymph node have been found to associate with early clinical stage and the absence of lymph node metastasis^66^. We found oophorectomy increased macrophages without altering M2 macrophages, thereby indicating M1 macrophages may have been impacted. We also found that estrogen increased the proportion of CD206 + macrophages and also decreased dendritic cells and T cells, which together would be considered pro-tumorigenic. The effects of endocrine therapies on the lymph node were more subtle, with oophorectomy causing an increase in macrophages (but not CD206 + M2 macrophages) and the proportion of central memory CD4 + T cells and central memory and naïve CD8 + T cells. Fulvestrant, on the other hand, increased CD69 + activated CD8 + T cells. CD4- CD8- double negative T cells were reduced by oophorectomy and fulvestrant treatment. Taken together, our work indicates that hormonal manipulation in the tumour naïve setting can impact the ability of cells in the mammary lymph node to phagocytose and present tumour cell debris/antigens. Future work assessing the spatial organisation of immune cells in the mammary gland and lymph node could help to define whether these changes are accompanied by localisation changes too.

## Methods

### Animal experiments

All animal work was conducted in accordance with the National Health and Medical Research Council guidelines under the approval of the Peter MacCallum Cancer Centre Animal Ethics Committee. The reporting of all animal experiments in the manuscript follows the recommendations in the ARRIVE guidelines. C57BL/6 mice were used to assess the effects of hormonal manipulation on the mammary immune microenvironment under homeostatic conditions. Mice were either left untreated, had their ovaries removed to eliminate endogenous estrogen and assessed 6 weeks later, or treated for 6 weeks with either a tumour stimulating dose of estrogen (17 $\beta$-estradiol, 0.3 mg pellet)^19^ or with the ER antagonist fulvestrant (5 mg/week i.p delivery). Mouse mammary glands and blood were then harvested for flow cytometric immune analysis as well as uterine horns as a measure of estrogen sensitivity. In the FACS experiments, n = 9–12 mice were used. For epithelial cytokine expression, we pooled samples for 4 biological replicates from n = 3 untreated control mice, n = 10 for fulvestrant treated mice, n = 2 for estrogen treated mice and n = 5 for oophorectomized mice. Different numbers of mice were used in an attempt to yield sufficient macrophages (including for RNA analysis), and macrophages are known to be reduced by fulvestrant treatment. Mice were not matched for the estrus cycle, but we have data in epithelial cell subpopulations to show the impact of the estrus cycle is negligible when compared to the impact of estrogen and anti-estrogen therapies.

### Mammary gland and Lymph node digestion to single cells

Mammary glands and draining lymph nodes were dissected from euthanized mice, separately isolated and collected in Leibovitz’s (L-15) medium (Gibco, USA) supplemented with 10% FBS (Gibco, USA) and 1% penicillin/streptomycin (Gibco, USA). Single cell dissociation was performed as previously described^67^. In brief, mammary glands and mammary lymph nodes were separately mechanically dissociated using a McIlwain tissue chopper (Mickle Laboratory Engineering, UK). Mammary glands were then digested in L-15 medium containing 1 mg/ml collagenase type 4 (Worthington, USA), 50 U/ml DNAse 1 (Roche, Switzerland) and 5 mM CaCl_2_ (Sigma-Aldrich, USA) for 1 h rotating at 37 °C. Lymph nodes did not require enzymatic digestion. Mammary glands and lymph nodes were both then subjected to centrifugation to achieve cell pellets of the respective tissues. Mammary gland and lymph node pellets were then each resuspended in 1 ml L-15. Cells of all tissues were sieved through a 100 mµ nylon strainer (Greiner Bio-One, Austria) to achieve a single cell suspension. Following an additional centrifugation, cells were resuspended in FACS buffer (consisting of PBS, 2% FBS, 2 mM EDTA and 15.3 µM sodium azide) in preparation for FACS antibody staining. All centrifugation steps were performed at 1500 rpm for 5 min at RT.

### Blood leukocytes

Blood was collected via cardiac puncture immediately following euthanasia. 1 ml syringes were used to draw blood before transfer into EDTA-coated collection tubes to prevent coagulation. Blood was centrifuged for 10 min at 10,000 rpm. The upper aqueous layer of clear plasma was collected and stored at – 80 °C for use in future assays. The remaining blood was resuspended in 10 ml red blood cell (RBC) lysis buffer to remove RBCs and incubated at RT for 5 min. Cells then underwent centrifugation followed by a second incubation in 5 ml RBC lysis buffer. After an additional centrifugation step, cells were washed in PBS. A final centrifugation step was performed before cells were resuspended in FACS buffer in preparation for flow cytometry antibody staining. Aside from the first centrifugation, all steps were performed at 1500 rpm for 5 min at RT.

### Flow cytometric immune analysis

Single cell suspensions from mammary glands and blood were stained in 50 µl FACS buffer on ice for 45 min. Mouse-specific primary antibodies were used to differentiate various subsets of immune cells. Antibody panel 1 differentiates cells of the innate immune system, whilst antibody panels 2 and 3 differentiate cells of the adaptive immune system (Supplementary Fig. 1, Adapted from previously published protocols^67^. Following staining, samples in panel 1 and 2 were centrifuged and preserved using IC Fixation Buffer (Invitrogen, USA) for 20 min before a final centrifugation and resuspension in FACS buffer prior to analysis. Samples stained with panel 3 required intracellular staining, so were preserved in Fixation-Permeabilization Buffer (Invitrogen, USA) for 20 min, then centrifuged and stained with the intracellular antibody for FOXP3 diluted in Permeabilization diluent (Invitrogen, USA) for 20 min. Cells were centrifuged before the final cell pellet was resuspended in FACS buffer for analysis (mammary glands in 200 µl, blood in 100 µl). All centrifugation steps were set at 524 g for 5 min at RT. Samples were analysed on the LSRFortessaTM X20 flow cytometer (BD Pharminogen, USA). Flow cytometry data was analysed using FlowLogic software. All FACS antibodies and clones are shown in Supplementary Table 1.

### Quantitative PCR analysis

Total RNA was extracted from FACS sorted cells by PicoPure RNA Isolation Kit (Arcturus Engineering), including DNAse treatment, as per the manufacturer’s instructions. Complementary DNA was synthesised from 10 ng of total RNA using the Superscript III First Strand Synthesis System for reverse transcriptase (RT)-PCR (Invitrogen) according to the manufacturer’s instructions. Quantitative PCR was carried out on a CFX384 Touch Real-Time PCR system (BioRad), using PowerUp SYBR Green Master Mix (Applied Biosystems) in a total volume of 5 ul. mRNA levels of target genes were normalized against the reference gene *Gapdh* using the method described by Pfaffl^68^. Primers are provided in Supplementary Table 2.

### Statistical analysis

All data was analysed using Prism v7 statistical software (GraphPad, USA). Results are expressed as mean $\pm$ SEM. All data was subjected to a D’Agostino-Pearson omnibus normality test to determine whether data was Guassian or non-Guassian in nature. Data was analysed using a Turkey multiple comparisons one-way ANOVA if a Gaussian distribution was confirmed, or a Kruskal–Wallis multiple comparisons one-way ANOVA if non-Gaussian. Any outlying data points were identified with a Grubbs test for outliers and excluded from further analysis. P values less than 0.05 were considered statistically significant.

## Supplementary Information

Supplementary Figure 1.

Supplementary Figure 2.

Supplementary Legends.

Supplementary Table 1.

Supplementary Table 2.

Supplementary Table 3.

## Data Availability

All data generated or analysed during this study are included in this published article and its supplementary information files.
