# Supplementary figures and images for: Estrogen-induced immune changes within the normal mammary gland
Source: Sci Rep. 2022 Nov 8;12:18986. doi: 10.1038/s41598-022-21871-4 (PMC9643548; doi:10.1038/s41598-022-21871-4)

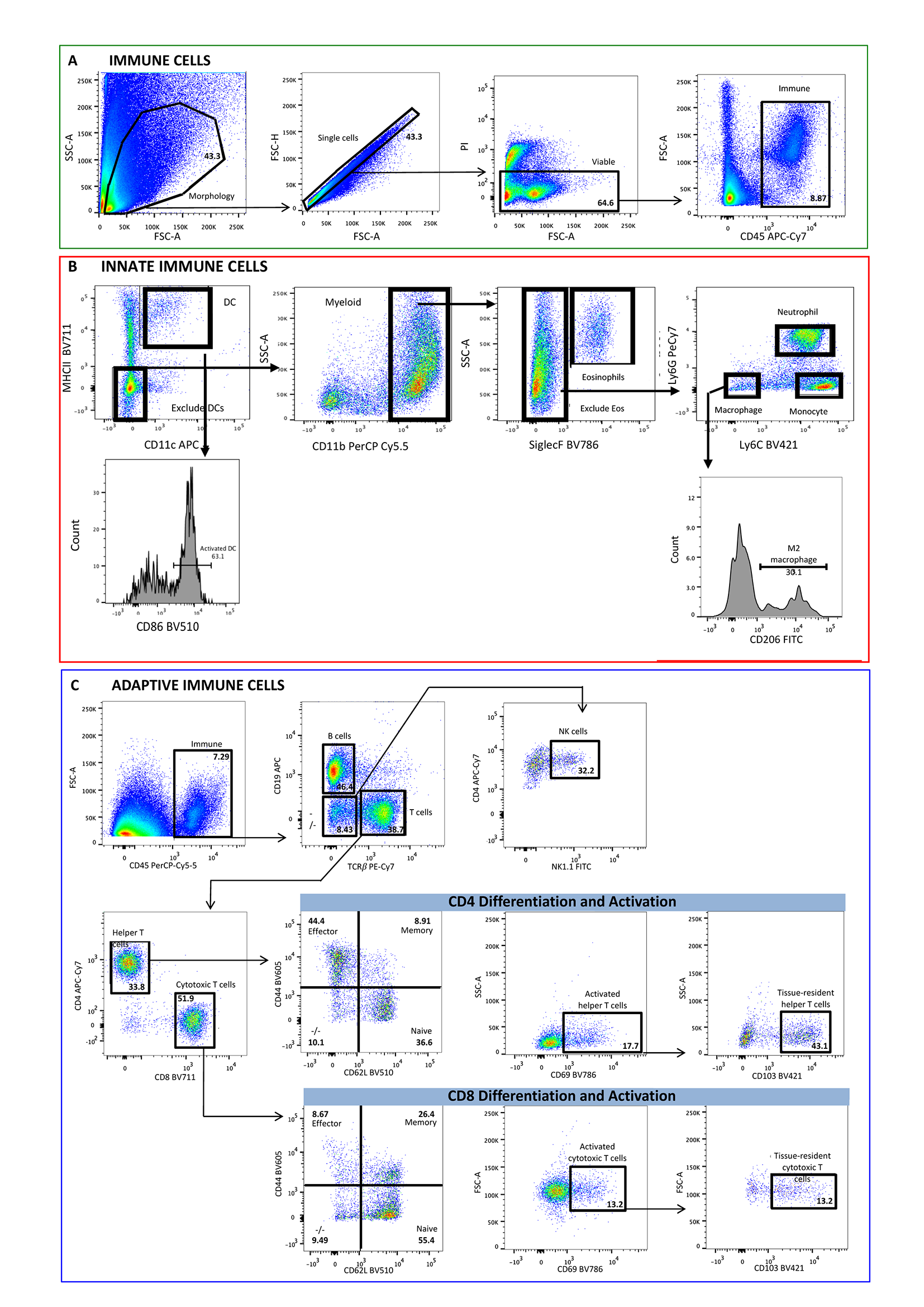

Supplement: Supplementary file 1 — Supplementary Figure 1. [file 41598_2022_21871_MOESM1_ESM.tif]

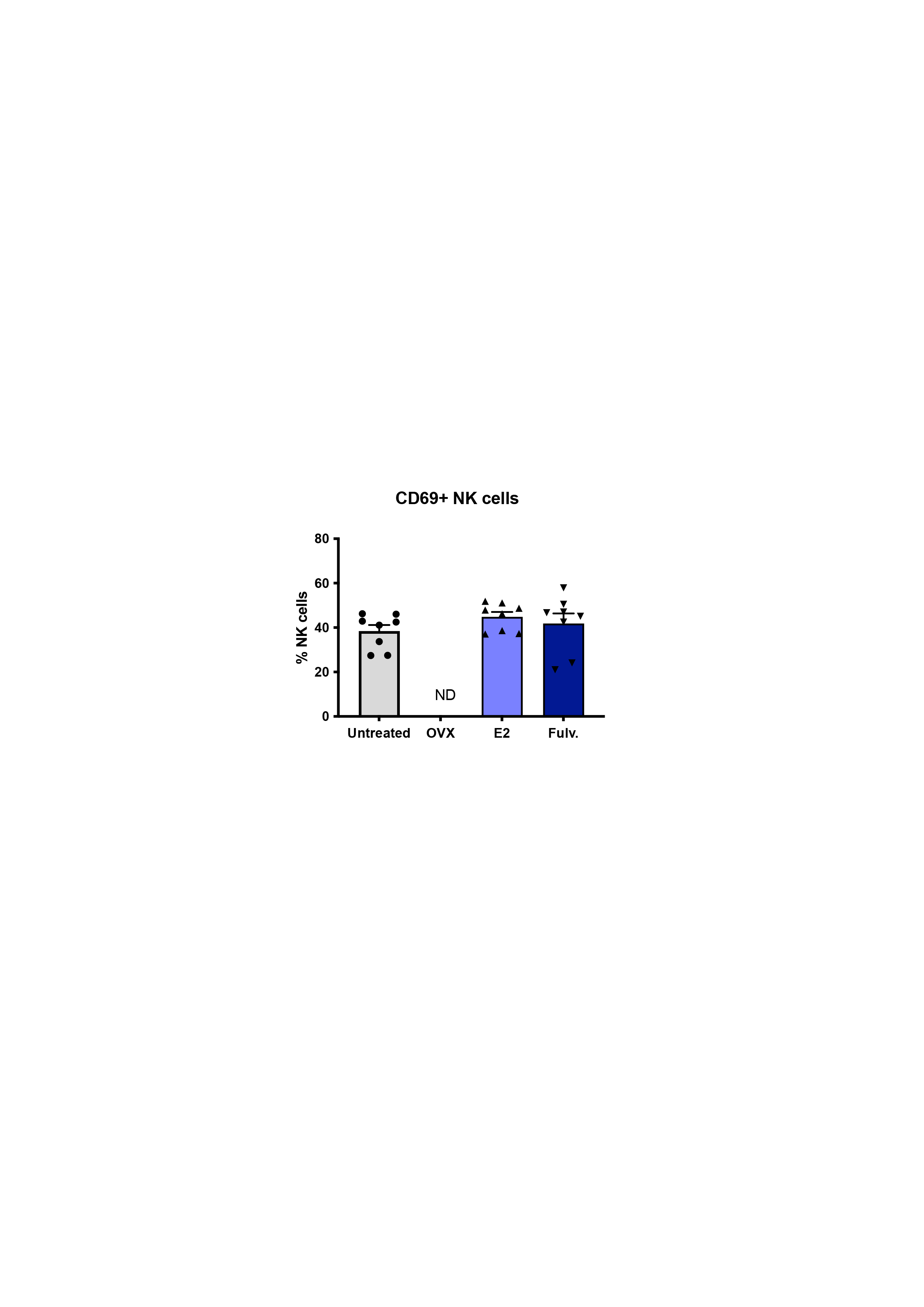

Supplement: Supplementary file 2 — Supplementary Figure 2. [file 41598_2022_21871_MOESM2_ESM.tif]
