# Supplementary material for: Estrogen-induced immune changes within the normal mammary gland
Source: Sci Rep. 2022 Nov 8;12:18986. doi: 10.1038/s41598-022-21871-4 (PMC9643548; doi:10.1038/s41598-022-21871-4)
Supplement: Supplementary file 3 — Supplementary Legends. [file 41598_2022_21871_MOESM3_ESM.docx]

**Supplementary Figure 1**: **Flow cytometry gating scheme for immune analysis of mammary glands, lymph nodes and blood.** Flow cytometry gating scheme for mouse mammary glands. Green box (**A**) shows how Leukocytes were gated according to morphology (SSC-A vs. FSC-A), doublets were excluded (FSC-H vs. FSC-A) and live immune cells were selected using PI and CD45. Red box (**B**) shows the isolation of the innate immune cells. Dendritic cells were identified and/or excluded from the myeloid cell analysis, by gating on CD11c and MHCII double positive cells. Myeloid cells were then identified as CD11b+. Eosinophils were identified and/or excluded from myeloid population before they were further dissected into macrophages (CD11b+ Ly6G- Ly6C^low^), monocytes (CD11b+ Ly6G-, Ly6C^high^), and neutrophils (CD11b+ Ly6G+ Ly6C^low^) using Ly6C vs. Ly6G. The alternatively activated macrophages (M2 macrophages) were subsequently identified as CD11b+ Ly6G-Ly6C^low^CD206+. Blue box (C) shows the isolation of the adaptive immune cells. A CD19 vs. TCRb plot was used to discriminate between T cells (TCRB+ CD19-) and B cells (TCRb- CD19+). The NK cells were gated from the double negative TCRB- CD19- population using NK1.1. CD4 vs. CD8a was then used to differentiate T cells into T helper cells (TCRb+CD19-CD4+CD8-) and cytotoxic T cells (TCRb+CD19-CD4-CD8+). F: The memory/effector status of both CD8 and CD4 T cells was determined using a CD62L vs. CD44 plot on both populations, to characterize naïve (CD62L+ CD44-), memory (CD62L+ CD44+), and effector (CD62L-CD44+). CD69 and CD103 were then used to isolated tissue resident memory CD4 (TCRb+CD19-CD4+CD8- CD69+CD103+) and CD8 (TCRb+CD19-CD4-CD8+CD69+CD103+) T cells. The gates shown are all derived from mouse blood, mammary gland or lymph node from either treated or untreated mice to show the populations with clarity.

**Supplementary Figure 2**: Effects of estrogen, and fulvestrant on the activation of NK cells as determined using the activation marker CD69. Mice were either untreated, or treated for 6 weeks with estrogen (E2) or Fulvestrant (Fulv). Results are expressed percentage of immune cells (mean ± SEM). Data was analysed using a Turkey multiple comparisons one-way ANOVA. Statistically significant results are denoted with *p<0.05. ND= no data for the oophorectomized group.
