# Supplementary material for: Estrogen-induced immune changes within the normal mammary gland
Source: Sci Rep. 2022 Nov 8;12:18986. doi: 10.1038/s41598-022-21871-4 (PMC9643548; doi:10.1038/s41598-022-21871-4)
Supplement: Supplementary file 4 — Supplementary Table 1. [file 41598_2022_21871_MOESM4_ESM.docx]

**Supplementary Table 1: Flow cytometry primary antibodies.**

| **Antibody name** | **Clone** | **Company** | **Host** |
| --- | --- | --- | --- |
| Panel 1 – Innate immune cells | | | |
| **CD45.2 APC-Cy 7** | 104 | BD Biosciences, USA | Mouse monoclonal |
| **CD11c APC** | HL3 | BD Biosciences, USA | Hamster monoclonal |
| **MHC BV711** | M5/114 | BD Biosciences, USA | Rat monoclonal |
| **CD11b PerCP-Cy 5.5** | M1/70 | BD Biosciences, USA | Rat monoclonal |
| **Ly6G PE-Cy 7** | 1A8 | BD Biosciences, USA | Rat monoclonal |
| **Ly6C BV421** | AL-21 | BD Biosciences, USA | Rat monoclonal |
| **CD206 FITC** | C068C2 | Biolegend, USA | Mouse monoclonal |
| **Siglec F BV786** | E50-2440 | BD Biosciences, USA | Rat monoclonal |
| **NKG2D PE** | CX5 | Invitrogen, USA | Mouse monoclonal |
| Panel 2 – Adaptive immune cells | | | |
| **CD45.2 PerCP-Cy 5.5** | 104 | BD Biosciences, USA | Mouse monoclonal |
| **CD19 APC** | 1D3 | BD Biosciences, USA | Rat monoclonal |
| **TCR**$\boldsymbol{\beta}$ **PE-Cy7** | H57-597 | BD Biosciences, USA | Hamster monoclonal |
| **CD4 APC-Cy7** | GK1.5 | BD Biosciences, USA | Rat monoclonal |
| **CD8 BV711** | 53-6.7 | BD Biosciences, USA | Rat monoclonal |
| **CD62L BV510** | MEL-14 | BD Biosciences, USA | Rat monoclonal |
| **CD44 BV605** | IM7 | BD Biosciences, USA | Rat monoclonal |
| **CD69 BV786** | H1.2F3 | BD Biosciences, USA | Hamster monoclonal |
| **CD103 BV421** | M290 | BD Biosciences, USA | Rat monoclonal |
| **NK1.1 FITC** | PK136 | BD Biosciences, USA | Mouse monoclonal |
| Panel 3 – Adaptive immune cells | | | |
| **CD45.2 PerCP-Cy 5.5** | 104 | BD Biosciences, USA | Mouse monoclonal |
| **CD19 BV421** | 1D3 | BD Biosciences, USA | Rat monoclonal |
| **TCR**$\boldsymbol{\beta}$ **PE-Cy7** | H57-597 | BD Biosciences, USA | Hamster monoclonal |
| **CD4 APC-Cy7** | GK1.5 | BD Biosciences, USA | Rat monoclonal |
| **CD8 BV711** | 53-6.7 | BD Biosciences, USA | Rat monoclonal |
| **FOXP3 FITC** | FJK-16s | Invitrogen, USA | Rat monoclonal |
