# Supplementary material for: Estrogen-induced immune changes within the normal mammary gland
Source: Sci Rep. 2022 Nov 8;12:18986. doi: 10.1038/s41598-022-21871-4 (PMC9643548; doi:10.1038/s41598-022-21871-4)
Supplement: Supplementary file 5 — Supplementary Table 2. [file 41598_2022_21871_MOESM5_ESM.docx]

| **Target** | **Forward primer (5'-3')** | **Reverse primer (5'-3')** |
| --- | --- | --- |
| *Gapdh* | GGTGCTGAGTATGTCGTGGA | GCTCTGGGCTTCATGGCGCT |
| *Tgfb1* | CTCCCGTGGCTTCTAGTGC | GCCTTAGTTTGGACAGGATCTG |
| *Ccl2* | GTTGGCTCAGCCAGATGCA | AGCCTACTCATTGGGATCATCTTG |
| Ccl5 | ATATGGCTCGGACACCACTC | CTTCGAGTGACAAACACGACTG |
| H2-K1 (MHCI) | CTTCGAGTGACAAACACGACTG | CTTCAGGTCTGCTGTGATGG |
| Ifng | ATGAACGCTACACACTGCATC | CCATCCTTTTGCCAGTTCCTC |
| Tnfa | ACCCTCACACTCAGATCATC | GAGTAGACAAGGTACAACCC |
| Csf1 | TGAGTCTGTCTTCCACCTGCT | CCCACAGAAGAATCCAATGTC |
| Csf2 | GGCCTTGGAAGCATGTAGAGG | GGAGAACTCGTTAGAGACGACTT |
| Il4 | CACAGGAGAAGGGACGCCATGC | ATGCGAAGCACCTTGGAAGCCC |
| Il10 | GGTTGCCCAGCCTTATCGGA | ACCTGCTCCACTGCCTTGCT |
| Il13 | GCTCTGGGCTTCATGGCGCT | AGGGCTACACAGAACCCGCCA |
