# Supplementary material for: Estrogen-induced immune changes within the normal mammary gland
Source: Sci Rep. 2022 Nov 8;12:18986. doi: 10.1038/s41598-022-21871-4 (PMC9643548; doi:10.1038/s41598-022-21871-4)
Supplement: Supplementary file 6 — Supplementary Table 3. [file 41598_2022_21871_MOESM6_ESM.docx]

**Supplementary Table 3: Organ weights**

| **Weights (mg)** | **Untreated** | **Ovx** | **E2** | **ICI** |
| --- | --- | --- | --- | --- |
| Uterine horns | 94.28 (1.8) | 34.5 (0.25)**** | 136.01 (2.6)** | 31.67 (1.05)**** |
| Mammary gland | 221.66 (2.87) | 462.32 (9.26)**** | 202.15 (3.57) | 269.74 (5.34) |
| M. Lymph nodes | 12.54 (0.24) | 17.09 (0.82) | 9.72 (0.13) | 14.88 (0.45) |

Mean (± SEM). n=8-14 mice/group. Data was analysed using a Kruskal-Wallis multiple comparisons 1-way ANOVA compared to untreated control. **p<0.01, ****p<0.0001.
